# Curcumin Mitigates Oxidative Damage in Broiler Liver and Ileum Caused by Aflatoxin B1-Contaminated Feed through Nrf2 Signaling Pathway

**DOI:** 10.3390/ani14030409

**Published:** 2024-01-26

**Authors:** Jingyang Zhang, Xue Sun, Xuehong Chai, Yihan Jiao, Jing Sun, Shenao Wang, Hao Yu, Xingjun Feng

**Affiliations:** College of Animal Science and Technology, Northeast Agricultural University, Harbin 150030, China; zhangjingyang304@163.com (J.Z.); 18800434060@163.com (X.S.); cxh990906@163.com (X.C.); yihanjiao11@163.com (Y.J.); sunjing1228228@163.com (J.S.); 18037637322@163.com (S.W.); h15562167945@163.com (H.Y.)

**Keywords:** mycotoxin, aflatoxin B1, curcumin, broiler, liver, ileum, antioxidant, Nrf2 signaling pathway

## Abstract

**Simple Summary:**

Aflatoxin B1 (AFB1) is widely present in animal feed as a mycotoxin, and it poses a serious threat to human and animal health. Curcumin (CUR) as a feed additive has been well documented for its antimicrobial, anti-inflammatory, antioxidant, and antiviral activities. The present experiment was designed to investigate the mitigating effect of CUR on the growth performance, liver, and intestinal health of broilers fed AFB1-contaminated diets. The results showed that CUR alleviated liver and intestinal damage in broilers caused by feeding AFB1-contaminated diets, and this damage was associated with the Nrf2 pathway. Notably, CUR also mitigated the changes in intestinal permeability caused by AFB1, which may be closely related to liver health (via the gut–liver axis). These results provide new ideas for future research on the therapeutic mechanism of CUR in alleviating AFB1 poisoning in broilers.

**Abstract:**

This experiment aimed to investigate the mitigating effect of CUR on the growth performance and liver and intestinal health of broilers fed AFB1-contaminated diets. In this study, 320 one-day-old healthy male Arbor Acres (AA) broilers were randomly divided into four groups, including the Control group (fed the basal diet), the AFB1 group (fed the AFB1-contaminated diet containing 1 mg/kg AFB1), the AFB1+CUR group (fed the AFB1-contaminated diet with 500 mg/kg CUR), and the CUR group (fed the basal diet containing 500 mg/kg CUR), with eight replicates of ten animals per group and a 28 d experimental period. In terms of the growth performance, the addition of 500 mg/kg CUR significantly improved AFB1-induced significant reductions in the final body weight on day 28 and mean daily gain (*p* < 0.05) and increased the ratio of the mean daily feed intake to mean daily weight gain in broilers (*p* < 0.05). In terms of liver health, significant improvements in liver histological lesions occurred in broilers in the AFB1+CUR group compared to the AFB1 group, with significantly higher glutathione peroxidase (GSH-Px), catalase (CAT), and total superoxide dismutase (T-SOD) activities (*p* < 0.05) and significantly higher levels of nuclear factor erythroid 2-related factor 2 (Nrf2), Kelch-like ECH-associated protein 1 (Keap-1), heme oxygenase 1 (HO-1), and NAD(P)H quinone oxidoreductase 1 (NQO-1) gene expression (*p* < 0.05). In terms of intestinal health, CUR addition significantly increased the relative length of ileum (*p* < 0.05), significantly elevated the height of ileal villi (*p* < 0.05), significantly reduced D-Lactate (D-LA) and diamine oxidase (DAO) activities in broiler serum (*p* < 0.05), significantly increased GSH, CAT, and T-SOD activities in ileal tissues (*p* < 0.05), and significantly elevated the expression of *Nrf2*, *HO-1*, and *NQO-1* genes (*p* < 0.05) compared to the AFB1 group. In conclusion, CUR showed a protective effect against damage to the liver and intestine caused by AFB1 in broilers through the Nrf2 signaling pathway, thereby improving the growth performance of broilers exposed to AFB1.

## 1. Introduction

The liver is the most common target organ of AFB1, and numerous studies have demonstrated its role in causing liver damage, potentially leading to cirrhosis and even liver cancer [1]. It has been reported that the toxic effects of AFB1 on the liver of animals are mainly manifested by jaundice, liver congestion, hepatocyte degeneration, and necrosis [2]. In addition to the liver, AFB1 has a strong toxic effect on the intestines [3,4], kidneys [5], and reproductive organs [6]. Oxidative stress has been widely recognized as a mechanism for AFB1 toxicity. Prolonged exposure to AFB1 stimulates the release of reactive oxygen species (ROS) by activated phagocytes, leading to increased oxidative levels, the inhibition of tissue self-repair functions, and subsequent oxidative damage [7]. Among them, various antioxidant enzymes in the body’s immune system and the Nrf2 signaling pathway are the keys to resisting the body from oxidative stress [7]. Poultry, especially chicks, is highly sensitive to AFB1 among livestock and poultry. Dietary AFB1 has been linked to liver swelling, necrosis, and severe vacuolar degeneration in broilers [8]. In addition, AFB1 promoted oxidative stress, increased the levels of ROS and MDA, and inhibited antioxidant enzyme activities as well as the Nrf2 signaling pathway in broiler livers [9]. The intestine, being the primary organ for AFB1 absorption, absorbs approximately 50% of AFB1 in the contaminated food. Previous studies have found that AFB1 induced an inflammatory response in the gut [10]. The study by Sarker et al. revealed that AFB1 impaired intestinal morphology, increased intestinal permeability, and inhibited the Nrf2 signaling pathway and the activity of antioxidant enzymes [11]. AFB1 was also found to promote oxidative stress in the broiler gut and disrupt the intestinal barrier in the study of Tao et al. [12]. Consequently, the damage to the liver and intestine of broilers by AFB1 is closely related to the Nrf2 signaling pathway, and the Nrf2 pathway is an important endogenous cellular mechanism for coping with oxidative stress [13].

CUR, as a polyphenolic compound, has a wide range of pharmacological activities, and its safety has been confirmed [14,15]. With its unique β-diketone moiety structure, CUR is considered a natural antioxidant [16]. CUR stimulates antioxidant enzyme activity, scavenges free radicals, inhibits ROS generation, blocks lipid peroxidation of cell membranes, and up-regulates antioxidant gene expression through the Nrf2 transcription factor, which results in the enhancement of the antioxidant functions of animals [17]. More importantly, studies have shown that CUR plays an important antioxidant role in protecting poultry health [18,19,20,21]. CUR has been found to improve the growth performance of broilers by enhancing intestinal morphology, subsequently affecting feed digestibility [22,23]. CUR has been found in vivo and in vitro studies to counteract the effects of negative factors on broiler health by promoting the expression of antioxidant enzymes through modulating the Nrf2 antioxidant pathway [24,25,26,27]. Notably, a large number of experiments have shown that oxidative stress in the body caused by AFB1 is an important cause of its damage to animal health [7].

Previous studies have shown that 1 mg/kg of AFB1 can lead to inflammatory liver damage in broilers [28], and in our previous study, the addition of 500 mg/kg of CUR to the feed inhibited oxidative stress in the liver and gut of poultry [21]. In this study, we investigated the damage to the liver and ileum of broilers caused by 1 mg/kg AFB1-contaminated diets, as well as the protective effect of 500 mg/kg CUR in diets, and focused on the role that the Nrf2 signaling pathway plays.

## 2. Materials and Methods

### 2.1. Chemicals

CUR with purity ≥ 98% (HPLC) (product number: Z100317) and AFB1 with purity ≥ 98% (HPLC) (product number: CASNO.1162–65-8) used in this study were purchased from Nanjing Jingzhu Biological Co., (Nanjing, China) and Shanghai Yuanye Bio-Technology Co., Ltd. (Shanghai, China), respectively.

### 2.2. Animal and Feeding Management

One-day-old male AA broilers were purchased from Weidong Broiler Breeder Production Specialist Co-operative in Harbin City, Heilongjiang Province. The experiment strictly adhered to the standards stipulated in the Regulations on the Management of Animal Testing in Heilongjiang Province (revised in 2016). All broilers were reared following the standard management of broiler with free access to food and water and routine immunization procedures.

### 2.3. Experimental Design

**Figure 1:**
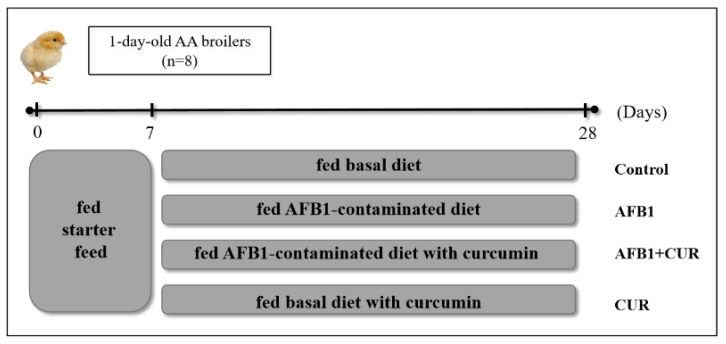
Study design for the feeding experiment.

A total of 320 1-day-old male AA broilers were randomly divided into four groups with 8 replicates of 10 animals per group. The four groups were including the Control group (fed a basal diet), the AFB1 group (fed an AFB1-contaminated diet containing 1 mg/kg AFB1), the AFB1+CUR group (fed an AFB1-contaminated diet with 500 mg/kg CUR), and the CUR group (fed a basal diet containing 500 mg/kg CUR), and the feeding experiment lasted for 28 d (Figure 1). The basal diets used in this experiment were configured to meet the nutritional requirements of the National Research Council (NRC, 1994). Chicks were fed starter feed from day 1 to day 7. The feed formulations for days 8 to 21 and 22 to 28 are shown in Table 1.

### 2.4. Sample Collection

On day 28, one chicken per replicate was selected for euthanasia according to American Veterinary Medical Association (AVMA) guidelines [29]. Broilers were bled after knockout stunning, and blood was collected from the broilers. The upper serum layer was collected after centrifugation at 3500 rpm 15 min at 4 °C and stored at −20 °C. Chickens were bled to death and the abdominal cavity was opened. The heart, liver, spleen, lungs, kidneys, pancreas, thymus, bursa of Fasciola, and intestine were removed intact. The surface fascia and adipose tissue were removed and weighed, while the length of each intestinal segment was measured. Two portions of liver and ileum tissues were taken, one portion was preserved in paraformaldehyde, and the other portion was quickly placed in liquid nitrogen and then transferred to −80 °C for preservation for subsequent analysis.

### 2.5. Organ Index Measurement

The formula calculate organ indices is as follows:

Organ index (%) = Organ weight (g)/Live weight (g) × 100

The relative length of each intestinal segment was calculated as follows:

Relative length (cm/kg·BW) = length of intestinal segment (cm)/live weight (kg)

### 2.6. Histological Analysis

Liver and ileal tissues fixed in paraformaldehyde were dehydrated and transparent. Three liver and ileal tissue samples were randomly selected per group for paraffin embedding. Subsequently, the embedded paraffin blocks were fixed and sectioned on a paraffin slicer with a thickness of 4 μm. Sections were dewaxed and hydrated for Hematoxylin Eosin (H&E) staining, dehydrated for transparency, and then sealed. Histopathological changes in the liver and ileum were examined under an optical microscope (Nikon Eclipse 80i, Tokyo, Japan). For the liver, at least three different fields of view were taken per section. For ileal tissue, villus height (VH) and crypt depth (CD) of at least three different microscopic fields were measured in each section using virtual microscope software (Image-Pro Plus 6.0). Three well-oriented villi and crypts were randomly selected on each field. The VH was measured from the tip to the bottom of the villi, and CD was the distance between its mouth and its base.

### 2.7. Determination of Antioxidant Indicators

Ileum and liver (about 100 mg) were devolved and mixed in a 0.9 mL stroke-physiological saline solution (4 °C, 0.9% NaCl, pH = 7.2–7.4) to obtain 10% tissue homogenate. The activity or content of GSH-Px, T-SOD, CAT, and MDA in the liver and ileum tissues were assessed according to the requirements of the GSH-Px kits (Item No.: A005-1-2), T-SOD kits (Item No.: A001-3-2), CAT kits (Item No.: A007-1-1), and MDA kit (Item No.: A003-1-2) (purchased from Nanjing Jiancheng Bioengineering Institute, Nanjing, China). The absorbance was determined using a UV–visible spectrophotometer (UV1100, MAPADA, Shanghai, China). The total protein content of each sample required in the kit was measured by the BCA Protein Assay Kit (Beyotime, Shanghai, China).

### 2.8. Determination of Intestinal Permeability

To assess the changes in the intestinal permeability of broilers treated in different groups, the levels of serum D-LA and DAO were determined using the ELISA kits (Shanghai Hengyuan Biological Co., Ltd., Shanghai, China).

### 2.9. Transcriptional Analyze

Total RNA was extracted from liver and ileum samples frozen at −80 °C using commercial Trizol reagent (Takara, Dalian, China). The concentration and purity of total RNA were examined by the A260/A280 ratio with a spectrophotometer (Implen Nanophotometer P-330, Munich, Germany). cDNA was synthesized by reverse transcription using Prime Script™ RT reagent kit with gDNA Eraser (catalog number: RR047A, Takara, Dalian, China). cDNA was amplified by real-time fluorescence quantitative PCR using TB Green™ Premix Ex Taq™ (catalog number: TB Green™ Premix Ex Taq™ (catalog number: RR086A, Takara, Dalian, China) kit. The primers for amplifying the genes *Nrf2*, *Keap1*, *HO-1*, *NQO1*, and *β-actin* were synthesized normally by Shanghai Shenggong Biotech. Co., and the sequences are shown in Table 2. The gene mRNA expression was calculated by the 2^−ΔΔCt^ method and normalized to the value of *β-actin*.

### 2.10. Statistical Analysis

All data were analyzed using one-way ANOVA with SPSS 26 (SPSS Inc., Chicago, IL, USA) software, and the experimental data were expressed as mean ± standard error of the mean (SEM), with LSD (one-way ANOVA, LSD) test as a post hoc test, and multiple comparisons of groups were performed using Duncan for significant differences, with *p* < 0.05 considered statistically significant. Significant differences were plotted using Excel and GraphPad Prism 8.0.1.

## 3. Result

### 3.1. Effect of CUR on the Growth Performance of Broilers Exposed to AFB1

The effects of the dietary addition of AFB1 and CUR on the growth performance of broilers are shown in Table 3. Compared with the Control group, the final body weight (28D BW) and average daily gain (ADG) were significantly (*p* < 0.05) lower, and the ratio of ADFI to ADG (F/G) was significantly (*p* < 0.05) higher at the ages of 1–28 days of broilers in the AFB1 group. For the 28D BW, ADG was significantly higher (*p* < 0.05), and F/G was significantly lower (*p* < 0.05) in the AFB1+CUR group compared to the AFB1 group. Compared with the Control group, 28D BW and ADFI were significantly higher (*p* < 0.05), and F/G was significantly lower (*p* < 0.05) in broilers aged 1-28 days in the CUR group.

### 3.2. Effect of CUR on the Organ Weight and Organ Indexes of Broilers Exposed to AFB1

The effect of the dietary addition of AFB1 and CUR on the organ weight and organ indexes of broilers is shown in Table 4. Compared with the Control group, the weights of the heart, liver, lung, pancreas, and bursa of broilers in the AFB1 group were significantly lower (*p* < 0.05), and the liver organ index was significantly higher. Compared with the AFB1 group, the weights of the heart, liver, lung, pancreas, and bursa of broilers in the AFB1+CUR groups were significantly higher (*p* < 0.05), with no significant changes in organ indexes. However, there was no significant difference in the liver organ index between the AFB1+CUR group and the Control group. There was no statistically significant change in the organ weight and organ index of broilers between the CUR and Control groups.

### 3.3. Effect of CUR on the Microstructure of Broiler Liver Exposed to AFB1

**Figure 2:**
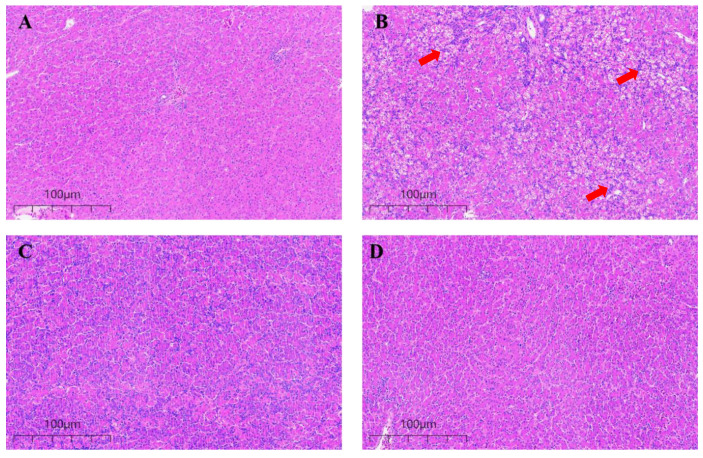
Histological examination broiler livers (bar = 100 µm). (**A**) Control group. (**B**) AFB1 group. (**C**) AFB1+CUR group. (**D**) CUR group. Vacuolar degeneration of hepatic cells in liver was indicated by red arrow in the AFB1 group (**B**).

Histological change results of liver tissue are shown in Figure 2. No abnormal morphology changes in liver cords and cells were observed in the Control group and the CUR group (Figure 2A,C). The liver tissue of the AFB1 group (Figure 2B) showed cellular swelling and vacuolar degeneration, irregular arrangement of hepatic cords, and infiltration of inflammatory cells. The cell structure and hepatic cord of the liver were obviously recovered in the AFB1+CUR group (Figure 2D) compared with the AFB1 group.

### 3.4. Effect of CUR on the Antioxidant Indicators of Broiler Liver Exposed to AFB1

**Figure 3:**
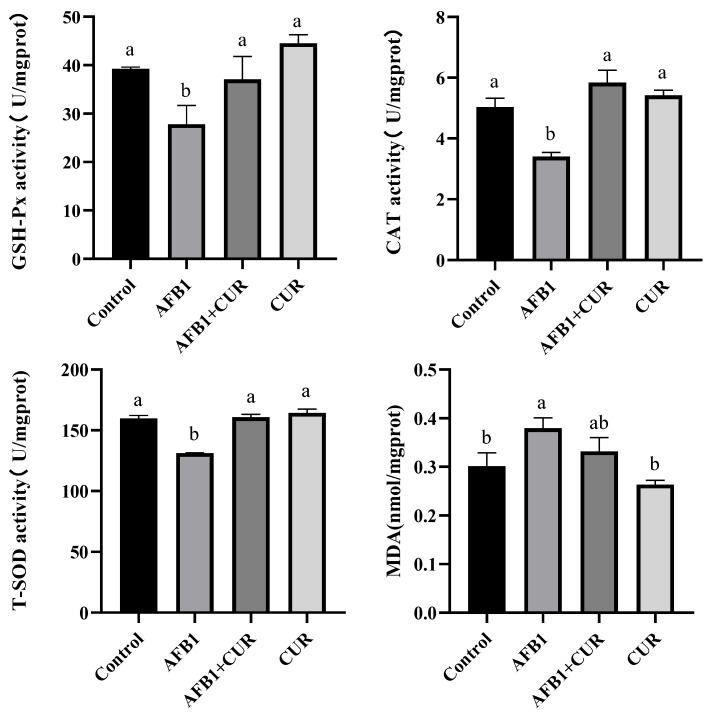
Effect of dietary addition of AFB1 and CUR on the antioxidant indicators of broiler livers exposed to AFB1. GSH-Px: glutathione peroxidase; CAT: catalase; T-SOD: total superoxide dismutase; MDA: malondialdehyde. ^a,b^ Values with different letter superscripts mean significant difference between the two groups (*p* < 0.05). Values are expressed as mean ± SEM.

The effect of the dietary addition of CUR on the antioxidant indicators of broiler livers exposed to AFB1 is shown in Figure 3. Compared with the Control group, the activities of GSH-Px, CAT, and T-SOD were significantly lower (*p* < 0.05), and the MDA content was significantly higher (*p* < 0.05) in the liver of broilers in the AFB1 group. Compared with the AFB1 group, GSH, CAT, and T-SOD activities were significantly higher (*p* < 0.05) after the addition of CUR in the AFB1-contaminated diet, but the MDA content showed no significant difference. There was no statistically significant change in the levels of GSH-Px, CAT, T-SOD, and MDA in liver of broilers between the CUR group and the Control group.

### 3.5. Expression Levels of Nrf2 Pathway Genes in Liver

**Figure 4:**
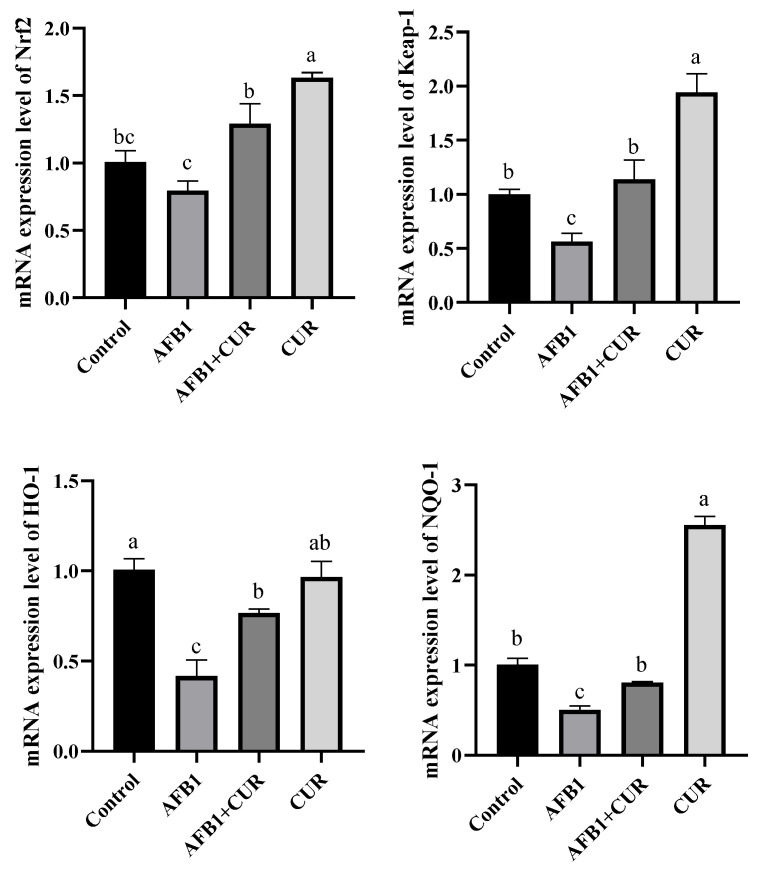
Effect of dietary CUR on the expression levels of Nrf2 pathway-related genes in broiler liver exposed to AFB1. *Nrf2*: nuclear factor erythroid2-related factor 2; *Keap-1*: Kelch-like ECH-associated protein 1; *HO-1*: heme oxygenase-1; *NQO-1*: NAD(P)H: quinone oxidoreductase 1. ^a,b,c^ Values with different letter superscripts mean significant difference (*p* < 0.05). Values are expressed as mean ± SEM.

The effect of dietary CUR on the expression levels of Nrf2 pathway-related genes in broiler liver exposed to AFB1 is shown in Figure 4. Compared with the Control group, the expression levels of *Keap-1*, *HO-1*, and *NQO-1* genes were significantly (*p* < 0.05) reduced in the liver of broilers in the AFB1 group, and there was a tendency for the Nrf2 gene expression level to be reduced, but not significantly. Compared with the AFB1 group, the mRNA levels of Nrf2, Keap-1, HO-1, and NQO-1 genes were significantly increased (*p* < 0.05) in the liver of broilers in the AFB1+ CUR group. The expression of *Nrf2*, *Keap-1*, and *NQO-1* genes in the liver of broilers in the CUR group was significantly higher (*p* < 0.05) compared to the Control group.

### 3.6. Effect of CUR on the Relative Weight and Length of Broiler Intestine Exposed to AFB1

The effect of the dietary CUR on the relative weight and length of each intestinal segment of the broilers exposed to AFB1 is shown in Table 5. In terms of intestinal weight, it was significantly lower (*p* < 0.05) in broilers in the AFB1 group compared to the Control group. Compared with the AFB1 group, the intestinal weight of broilers in the AFB1+CUR group was significantly increased (*p* < 0.05). There was no significant difference in the intestinal weight of broilers between the CUR group and the Control group. There was no significant difference in the relative weight of the intestine between the four groups. The relative length of the duodenum, jejunum, and ileum in the broilers in the AFB1 group was significantly lower (*p* < 0.05) than those in the Control group. Compared with the AFB1 group, the relative length of the ileum of broilers in the AFB1+CUR group was significantly higher (*p* < 0.05), while there was no significant change in the relative length of the duodenum and jejunum. There was no significant difference in the intestinal relative length of broilers between the CUR group and the Control group.

### 3.7. Effect of CUR on the Ileum Morphology of Broilers Exposed to AFB1

**Figure 5:**
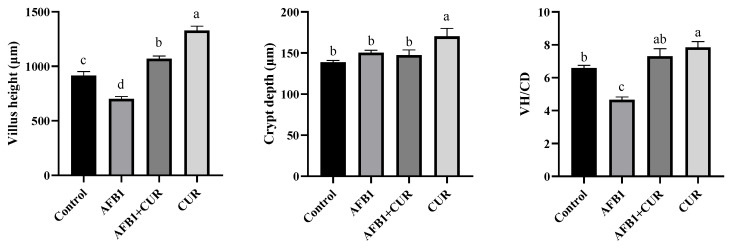
Effect of the dietary CUR on the ileal morphology in broilers exposed to AFB1. ^a,b,c,d^ Values with different letter superscripts mean significant difference (*p* < 0.05). Values are expressed as mean ± SEM.

The length of the villi and the depth of the crypts in the ileum were measured based on the histological examination of the broiler ileum (Supplementary Figure S1). As shown in Figure 5, the villus length in the ileum and the ratio of the villi length to crypt depth (VH/CD) of broilers in the AFB1 group were significantly lower (*p* < 0.05) than those in the Control group, and there was no significant change in the depth of crypts. Compared with the AFB1 group, the addition of CUR to AFB1-contaminated diets significantly increased the villus length and VH/CD in the AFB1+CUR group (*p* < 0.05), but it caused no significant change in crypt depth. The villus length, the depth of crypts, and the VH/CD of broiler ileum in the CUR group were significantly higher (*p* < 0.05) than those in the Control and AFB1 groups.

### 3.8. Effect of CUR on the Intestinal Permeability of Broilers Exposed to AFB1

**Figure 6:**
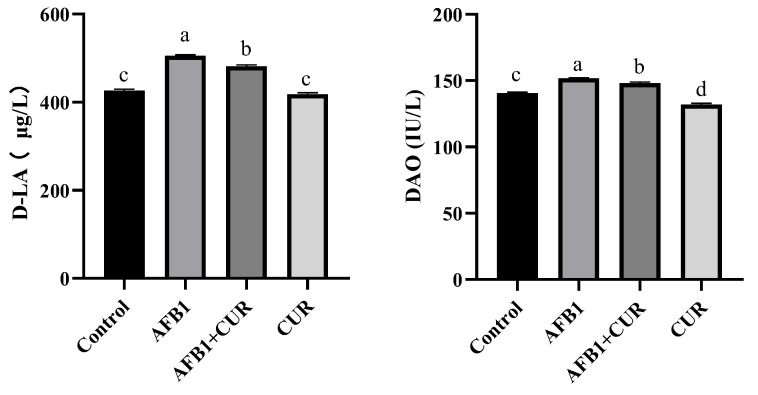
Effect of the dietary CUR on intestinal permeability in broilers exposed to AFB1. D-LA: D-Lactate; DAO: diamine oxidase. ^a,b,c,d^ Values with different letter superscripts mean significant difference (*p* < 0.05). Values are expressed as mean ± SEM.

To assess the effect of the dietary addition of CUR on the intestinal permeability in broilers exposed to AFB1, the D-LA and DAO levels in the serum were determined. As shown in Figure 6, both D-LA and DAO levels in the serum of broilers in the AFB1 group were significantly higher (*p* < 0.05) than those in the Control group. Compared with the AFB1 group, the addition of CUR to AFB1-contaminated diets significantly decreased both D-LA and DAO levels in the serum (*p* < 0.05). Compared with the Control group, the DAO activity in broiler serum in the CUR group was significantly reduced (*p* < 0.05), while the serum D-LA level showed no significant change.

### 3.9. Effect of CUR on the Antioxidant Indicators of Broiler Ileum Exposed to AFB1

**Figure 7:**
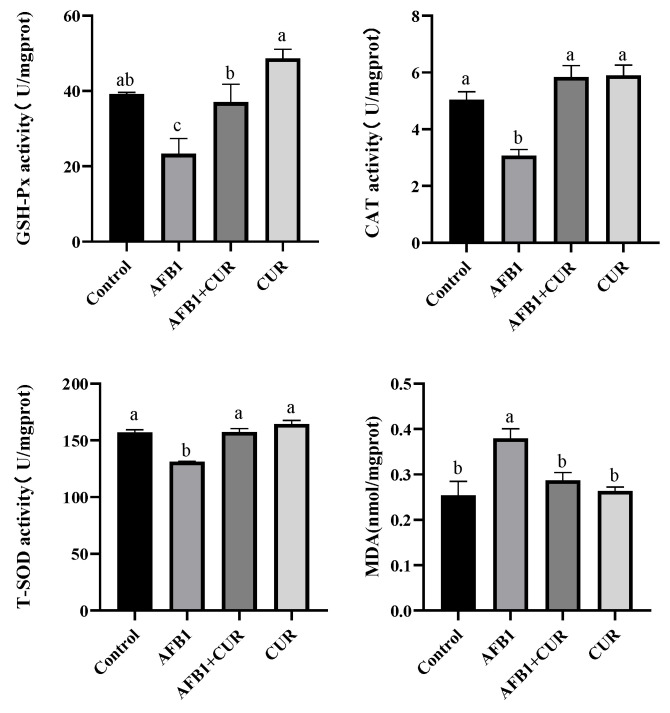
Effect of dietary CUR on the antioxidant indicators of broiler ileum exposed to AFB1. GSH-Px: glutathione peroxidase; CAT: catalase; T-SOD: total superoxide dismutase; MDA: malondialdehyde. ^a,b,c^ Values with different letter superscripts mean significant difference (*p* < 0.05). Values are expressed as mean ± SEM.

As shown in Figure 7, compared with the Control group, GSH-Px, CAT, and T-SOD activities were significantly reduced (*p* < 0.05), and the MDA content was significantly increased (*p* < 0.05) in the ileal tissue of broilers in the AFB1 group. GSH, CAT, and T-SOD activities were significantly higher (*p* < 0.05), and the MDA content was significantly lower (*p* < 0.05) in the ileal tissue of broilers in the AFB1+ CUR group than those in the AFB1 group. There was no significant change in GSH, CAT, T-SOD, and MDA in the ileal tissue of broilers between the CUR group and the Control group.

### 3.10. Effect of CUR on the Expression of Nrf2 Pathway Genes in Broiler Ileum Exposed to AFB1

**Figure 8:**
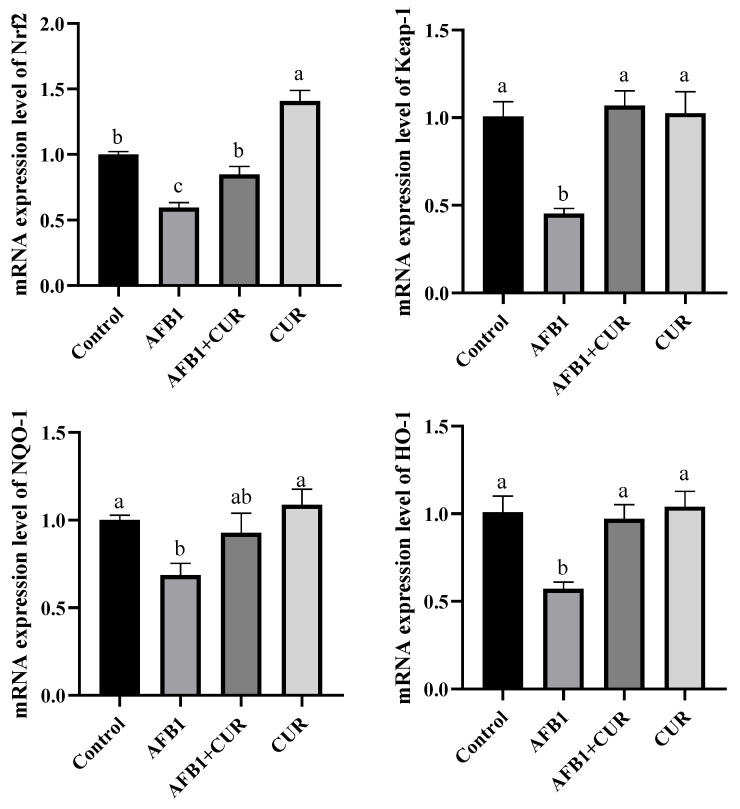
Effect of dietary CUR on the expression levels of Nrf2 pathway-related genes in broiler ileum exposed to AFB1. Nrf2: nuclear factor erythroid2-related factor 2; Keap-1: Kelch-like ECH-associated protein 1; HO-1: heme oxygenase-1; NQO-1: NAD(P)H: quinone oxidoreductase-1. ^a,b,c^ Values with different letter superscripts mean significant difference (*p* < 0.05). Values are expressed as mean ± SEM.

As shown in Figure 8, compared with the Control group, the mRNA levels of *Nrf2*, *Keap-1*, *HO-1*, and *NQO-1* genes were significantly reduced (*p* < 0.05) in the ileal tissue of broilers in the AFB1 group. The AFB1+CUR group showed significantly higher mRNA levels of *Nrf2*, *Keap-1*, *HO-1*, and *NQO-1* genes in the ileal than the AFB1 group (*p* < 0.05). The mRNA level of the Nrf2 gene in the ileum of broilers in the CUR group was significantly higher (*p* < 0.05) than that in the Control group, while there was no significant difference in the mRNA levels of the *Keap-1*, *NQO-1*, and *HO-1* genes between the two groups.

## 4. Discussion

In this study, feeding contaminated feeds with AFB1 at a concentration of 1 mg/kg caused a reduced growth performance in broilers (Table 3), which is consistent with the results of similar previous studies. The results demonstrated that after 28 days of feeding AFB1-contaminated diets, broilers exhibited significantly lower 28D BW and ADG, a tendency toward lower ADFI, and significantly higher F/G compared to the Control group. These observations may be attributed to the poor palatability of AFB1-contaminated diets and the inhibition of organismal adipogenesis, along with the disruption of intestinal health caused by AFB1. AFB1 has been shown to cause depression, anorexia, and diarrhea in animals, which can lead to reduced body weight and feed intake [30]. Moreover, AFB1 alters lipid metabolism, which reduces the body’s growth performance by limiting lipogenesis and promoting lipolysis [31]. The specific mechanism may be that AFB1 can lead to dysregulation of fat metabolism by affecting the phosphatidylinositol 3 kinase (PI3K)/protein kinase B (AKT) signaling pathway [32]. For the F/G, it has been demonstrated that AFB1 causes damage to the intestinal barrier and reduces the activity of pancreatic enzymes, amylase, and trypsin, affecting the efficiency of food absorption [33]. Previous studies have shown that the addition of CUR to feed at a certain concentration promotes the growth performance of broilers, which is consistent with the findings of the present study [34,35]. The addition of CUR to AFB1-contaminated diets significantly increased 28D BW and ADG and decreased F/G in broilers compared to the AFB1-contaminated diet group, and the addition of CUR alone resulted in a significant upward trend of 28D BW and ADG and a significant decrease in F/G in broilers compared to the Control group. The above results indicate that the addition of CUR to feed can significantly improve the growth performance of broilers and alleviate the reduced growth performance of broilers caused by AFB1.

The liver is an important target organ of AFB1. The results of this experiment (Table 4) show that AFB1 caused a significant decrease in the liver weight and an increase in the liver index in broilers compared with the Control group. Previous studies have shown that AFB1 causes an increase in the liver index, which may be due to the addition of AFB1 to the liver, resulting in inflammation and enlargement [36]. To further investigate the effects of AFB1 and CUR on the liver, we performed pathological observations on liver tissue (Figure 2). The H&E-stained sections showed that AFB1 caused severe lesions in the liver tissue of broilers, with irregular arrangements of the hepatic cords and vacuolization of the liver tissue. When the liver tissue is damaged, vacuolization of the hepatocytes occurs. This is due to an increase in the water content in the damaged liver cells, which causes swelling and cytoplasmic laxity of the hepatocytes and further development of hepatocytes that are swollen and spherical, with almost transparent cytoplasm [37]. These phenomena proved that 1 mg/kg of AFB1 caused liver damage in broilers. The above results are consistent with the results of similar previous studies [38]. It is noteworthy that the liver tissue lesions underwent significant improvements after the addition of CUR, and the liver tissue sections in the CUR group showed a more regular and clearer arrangement of the hepatic cords compared with the Control group. These results illustrate that the addition of CUR to the feed can alleviate the liver damage caused by AFB1 in broilers and have a significant protective effect on the liver. Oxidative stress is an important contributor to pathological diseases that are caused by disturbances in the intrinsic redox system due to reactive oxygen species (ROS) [39]. In the present study, the AFB1-contaminated diet significantly reduced GSH-Px, CAT, and T-SOD activities, significantly increased the MDA content, and significantly inhibited the gene expression of *Keap-1*, *NQO-1*, and *HO-1* in the Nrf2 signaling pathway in broiler livers. This was the same discovery as Salem et al. [40] and Li et al. [9]. AFB1 poisoning leads to an increase in ROS production, which affects the expression of the antioxidant enzyme gene in the body [3,41,42]. MDA is the end product of lipid peroxides under ROS pressure and is a good indicator for assessing oxidative stress. SOD is a specific antioxidant enzyme that removes oxygen radicals. CAT is found in abundance in the liver and can effectively protect cells from oxidative damage. GSH-Px has an important role in resisting hepatic tissues from AFB1 and protecting normal cellular structure and function. Therefore, the content of MDA and the activities of the antioxidant enzymes SOD, CAT, and GSH are important for determining the severity of AFB1 poisoning [7]. In the past decade, the Nrf2 signaling pathway has been identified as an important endogenous cellular mechanism for coping with oxidative stress and regulating the activities of antioxidant enzymes in the body [13,43]. ROS can modify specific cysteine residues in Keap1 of the Keap1-Nrf2 complex in the cytoplasm, causing conformational changes of the complex and Nrf2 dissociation. The dissociated Nrf2 translocates into the nucleus and binds to the sMAF protein to form heterodimers, which then binds to the antioxidant response element (ARE) on chromosomes to induce the expression of a series of cytoprotective genes, such as *NQO1*, *HO-1*, etc. [44]. The addition of 500 mg/kg CUR to AFB1-contaminated feed restored the activities of antioxidant enzymes, including GSH-Px, CAT, and T-SOD in the livers of the broilers. As far as the Nrf2 signaling pathway is concerned, the CUR group significantly increased mRNA levels of several genes in this pathway (*Nrf2*, *Keap-1*, and *NQO-1*). In addition, the expression levels of the Nrf2-pathway-related genes (*Nrf2*, *Keap-1*, *HO-1*, and *NQO-1*) were significantly promoted in the AFB1+CUR group compared with the AFB1 group. So, the addition of 1 mg/kg of AFB1 to the feed caused oxidative damage to the broiler liver, which was closely related to the Nrf2 signaling pathway. The addition of 500 mg/kg CUR to the feed alleviated the oxidative damage caused by AFB1 to the broiler liver by promoting the Nrf2 signaling pathway.

The intestine, as the first organ to come into contact with AFB1 in a diet, is another major organ besides the liver that is facing damage from an AFB1 attack. In recent years, more and more attention has been paid to intestinal health and the relationship between the liver and the gut. A growing body of research has demonstrated that damage to the gut can negatively affect the liver [35,36]. When the intestinal barrier is compromised, some pathogenic factors (e.g., LPS) can pass through the intestinal barrier and reach the liver through the blood circulation, causing liver damage [37]. Therefore, the intestinal damage of AFB1 in broilers was investigated in this study.

AFB1 significantly reduced the weight of the intestine in broilers on day 28, and the addition of CUR reversed this reduction, with no change in the relative weight of the intestine (Table 5) due to the reduction in the broiler body weight caused by AFB1. The relative length of the duodenum, jejunum, and ileum was significantly reduced by AFB1, and a significant increase in the relative length of the ileum was caused by CUR. This suggested that increasing the relative length of the gut the amount could improve feed conversion efficiency.The small intestine is the main site of digestion and absorption of nutrients and the regulation of immunity and intestinal flora, which is the first line of defense against pathogenic bacteria and toxins [45]. The ileum, one of the important segments of the small intestine, also has important digestive, absorptive, and secretory functions [46,47]. The height of the villi (VH) and the depth of the crypts (CD) directly affect the absorption capacity of the intestine. A shorting of VH indicates a decrease in the absorption capacity of the intestine, while CD reflects the colonization rate and maturity of the crypt cells. The ratio of VH/CD is a comprehensive reflection of the digestive and absorption functional states of the small intestine [48]. Previous studies have illustrated that AFB1 caused damage to intestinal morphology, including a reduction in VH, CD, and VH/CD [49]. In this study, VH and CD were measured in histopathological sections of the ileum and the results showed (Figure 5) that AFB1-contaminated feed resulted in a significant reduction in VH and VH/CD in the broiler ileum, which suggested that AFB1 caused alterations in ileal morphology and reduced the digestive and absorptive capacity of the broiler ileums. CUR significantly increased VH and VH/CD in the ileum compared to the AFB1 group. Surprisingly, there was no difference in ileal VH, CD, and VH/CD between the AFB1+CUR group and the Control group, suggesting that CUR completely mitigated the negative effects of AFB1 on ileal morphology. In addition, compared with the Control group, the VH, DCs, and VH/CD were significantly increased in the CUR group, which indicated that CUR had a promoting effect on the absorptive capacity of the ileum and could enhance the absorption of nutrients in the ileum. Sarker et al. found that AFB1 disrupted the intestinal barrier in broilers [23]. Damage to the intestinal barrier can lead to increased intestinal permeability. Bacteria and their metabolites, endotoxins (LPS), and a variety of inflammatory factors can pass through the damaged intestinal barrier and reach the liver through the portal vein and systemic circulation, causing hepatic inflammation liver injury. Diamine oxidase (DAO) is an enzyme in the intestinal epithelium and will enter the blood when the intestinal barrier is disrupted [50]. D-LA, a metabolite of bacterial fermentation, is rarely absorbed due to intestinal barrier. When the intestinal barrier is damaged, D-LA will pass through the damaged intestinal mucosa and enter the blood [51]. Therefore, serum levels of DAO and D-LA are considered indicators to assess intestinal permeability. The results of this study (Figure 6) showed that the serum levels of D-LA and DAO in broilers were significantly increased after the addition of AFB1 to the feed, which indicated that AFB1 caused intestinal barrier damage in broilers. After the addition of CUR, the concentrations of D-LA and DAO were significantly reduced compared with those of the AFB1 group, suggesting that CUR attenuated the damage to the intestinal barrier of broilers caused by AFB1. Notably, DAO and D-LA were significantly lower in the CUR group compared to the Control group, suggesting that CUR has a promoting effect on the integrity of the intestinal barrier. Antioxidant enzymes and the Nrf2 signaling pathway play an important role in maintaining intestinal barrier integrity. When the intestinal antioxidant system is insufficient to resist the production of ROS, this is accompanied by changes in intestinal morphology and barrier disruption [52,53]. A study of Rajbir et al. demonstrated that activation of the Nrf2 pathway upregulated the contents of the tight junction proteins in the gut, thereby maintaining the barrier role of the gut [54]. In this study, the activities of antioxidant enzymes and the expression of Nrf2-signaling-pathway-related genes were determined in ileal tissues (Figure 7 and Figure 8). The results showed that feeding AFB1-contaminated diets resulted in a significant decrease in GSH-Px, CAT, and T-SOD activities, a significant increase in MDA content in broiler ileal tissues, and a significant suppression of the expression levels of the *Keap-1*, *NQO-1*, and *HO-1* genes in the Nrf2 signaling pathway. The addition of 500 mg/kg CUR to AFB1-contaminated feed reversed the changes in GSH-Px, CAT, T-SOD, and MDA levels in broiler ileal tissues induced by AFB1, in which there was no significant difference compared to the Control group. This suggests that the addition of CUR ameliorated the oxidative damage caused by AFB1. By comparing the CUR group and Control group, as well as the AFB1+CUR group and AFB1 group, it was found that the gene expression levels of *Nrf2*, *Keap-1*, *HO-1*, and *NQO-1* were significantly increased after CUR treatment, suggesting that the protective effect of CUR on the intestinal barrier may be achieved through the modulation of the Nrf2 signaling pathway.

## 5. Conclusions

In conclusion, the addition of 500 mg/kg CUR to the feed significantly ameliorated the negative effects of AFB1 (1 mg/kg) on broiler growth performance. Further analysis of the liver and intestine revealed that the addition of 500 mg/kg CUR to the feed reduced the liver and intestine damage and oxidative stress caused by AFB1 in broilers, and this ameliorative effect was related to the Nrf2 signaling pathway. CUR reduced the changes in the intestinal permeability of broilers induced by AFB1, which may be the reason for the alleviation of AFB1-induced liver damage in broilers by CUR. However, the exact mechanism needs to be further investigated.

## Figures and Tables

**Table 1:** Composition of experimental diets.

Ingredients, %	Days 7 to 21	Days 22 to 28
Corn	58.5	61.15
Soybean meal	30.0	26.3
Corn protein meal	4.06	4.33
Soybean oil	2.70	3.80
Limestone	1.33	1.26
Calcium bicarbonate	1.60	1.52
L-Lysine, 99%	0.20	0.14
DL-methionine, 98%	0.21	0.10
Sodium chloride	0.30	0.30
Choline chloride	0.10	0.10
Premix ^1^	1.00	1.00
**Nutrient levels**		
ME (MJ/kg)	12.54	12.96
Crude protein (%)	21.50	20.09
Calcium (%)	1.06	0.91
Total phosphorus (%)	0.73	0.69
Effective phosphorus	0.45	0.43
Lys (%)	1.15	1.01
Met (%)	0.55	0.43
Met+ Cys (%)	0.91	0.77
Thr (%)	0.80	0.73

^1^ Provided per kg of complete diet: Vitamin A, 12,000-IU; Vitamin D3, 2500-IU; Vitamin E, 20-IU; Vitamin K3, 1.3 mg; Thiamine, 2.2 mg; Riboflavin, 8.0 mg; Niacinamide, 40 mg; Calcium Pantothenate, 10 mg; Pyridoxine, 4 mg; Biotin, 0.04 mg; Folic Acid, 1 mg; Vitamin B12, 0.013 mg. Iron (from ferrous sulfate), 80 mg; copper (from copper sulfate), 80 mg; manganese (from manganese sulfate), 110 mg; zinc (from zinc sulfate), 60 mg; iodine (from calcium iodate), 11 mg; selenium (from sodium selenite), 0.3 mg.

**Table 2:** Primers used for relative real-time PCR.

Genes	Primer Sequence (5′ → 3′)	Product Size (bp)	Accession Number
*Nrf2*	F: GGGACGGTGACACAGGAACAAC	93	XM_046921130.1
	R: TCCACAGCGGGAAATCAGAAAGATC		
*Keap1*	F: CGCTTTCTTCAGGGGTAGCA	170	NM_205117.2
	R: AGTTCGGTGCAGAAGAGGTG		
*HO-1*	F: GCTGGGAAGGAGAGTGAGAGGAC	107	XM_046921508.1
	R: GCGACTGTGGTGGCGATGAAG		
*NQO1*	F: CGAGTGCTTTGTCTACGAGATGGAG	102	NM_001277620.2
	R: AGGTCAGCCGCTTCAATCTTCTTC		
*β-actin*	F: CTGTGCCCATCTATGAAGGCTA	139	NM_205518.2
	R: ATTTCTCTCTCGGCTGTGGTG		

**Table 3:** Effects of dietary addition of AFB1 and CUR on the growth performance of broilers.

Items	Treatments				SEM	*p*-Value
	Control	AFB1	AFB1+CUR	CUR		
1 D BW (g)	38.50	38.50	37.50	38.67	0.333	0.355
28 D BW (g)	936.82 ^b^	714.14 ^d^	849.00^c^	1001.59 ^a^	22.663	<0.001
1-28D						
ADG (g/bird)	32.08 ^b^	24.13 ^d^	29.00 ^c^	34.39 ^a^	0.808	<0.001
ADFI (g/bird)	48.60 ^ab^	46.85 ^c^	48.14 ^bc^	50.08 ^a^	0.342	0.003
F/G (g/g)	1.52 ^c^	1.94 ^a^	1.66 ^b^	1.46 ^d^	0.040	<0.001

1 D BW, 28D BW, ADG, ADFI, and F/G represent the means of 8 replicates. 1D BW = 1-day-old broiler weight; 28D BW = 28-day-old broiler weight; ADG = average daily weight gain; ADFI = average daily feed intake. ^a,b,c,d^ Values with different letter superscripts within the same row mean significant difference (*p* < 0.05). SEM = standard error of means.

**Table 4:** Effect of dietary addition of AFB1 and CUR on the organ indices of broilers.

Items	Treatments				SEM	*p*-Value
	Control	AFB1	AFB1+CUR	CUR		
Heart weight (g)	5.91 ^a^	4.61 ^b^	5.63 ^a^	63.88 ^a^	0.179	0.001
Heart index (%)	0.64	0.69	0.61	0.63	0.016	0.318
Liver weight (g)	20.66 ^a^	15.93 ^b^	21.13 ^a^	20.46 ^a^	0.546	<0.001
Liver index (%)	2.23 ^b^	2.44 ^a^	2.28 ^ab^	2.15 ^b^	0.038	0.042
Spleen weight (g)	0.99	0.84	1.01	0.98	0.042	0.473
Spleen index (%)	0.11	0.13	0.11	0.10	0.005	0.284
Lungs weight (g)	6.20 ^a^	4.20 ^b^	5.80 ^a^	5.89 ^a^	0.210	<0.001
Lungs index (%)	0.67	0.62	0.63	0.59	0.017	0.358
Kidney weight (g)	5.20	4.56	5.46	5.44	0.223	0.468
Kidney index (%)	0.57	0.68	0.59	0.54	0.028	0.286
Pancreas weight (g)	2.73 ^a^	1.93 ^b^	2.65 ^a^	2.63 ^a^	0.083	<0.001
Pancreas index (%)	0.30	0.29	0.29	0.26	0.007	0.345
Thymus weight (g)	2.95	1.87	3.03	3.31	0.056	0.003
Thymus index (%)	0.32	0.27	0.33	0.32	0.014	0.438
Bursa of Fabricius weight (g)	1.85 ^a^	1.00 ^b^	1.76 ^a^	1.88 ^a^	1.846	0.005
Bursa of Fabricius index (%)	0.20	0.15	0.19	0.19	0.203	0.371

^a,b^ Values with different letter superscripts within the same row mean significant difference (*p* < 0.05). SEM = standard error of means.

**Table 5:** Effect of the dietary CUR on the relative weight and length of each intestinal segment of broilers exposed to AFB1.

Items	Treatments				SEM	*p*-Value
	Control	AFB1	AFB1+CUR	CUR		
Intestinal weight and relative weight						
Intestine weight (g)	45.27 ^a^	35.30 ^b^	45.47 ^a^	48.17 ^a^	1.273	<0.001
Intestine index (%)	4.91	5.08	4.92	4.73	0.085	0.562
Relative length(cm/kg·BW)						
Duodenum relative length	19.73 ^a^	17.79 ^b^	18.27 ^b^	19.75 ^a^	0.239	<0.001
Jejunum relative length	54.69 ^a^	47.01 ^b^	51.44 ^ab^	54.45 ^a^	1.11	0.038
Ileum relative length	54.07 ^ab^	47.53 ^c^	51.33 ^b^	55.79 ^a^	0.87	<0.001

^a,b,c^ Values with different letter superscripts within the same row mean significant difference (*p* < 0.05). SEM = standard error of means.

## Data Availability

The data presented in this study are available on request from the corresponding author.

## Supplementary Materials

The following supporting information can be downloaded at https://www.mdpi.com/article/10.3390/ani14030409/s1, Figure S1: Histological examination broiler ileum (Bar = 200 µm). (A) Control group. (B) AFB1 group. (C) AFB1+CUR group. (D) CUR group.
