# Supplementary material for: Curcumin Mitigates Oxidative Damage in Broiler Liver and Ileum Caused by Aflatoxin B1-Contaminated Feed through Nrf2 Signaling Pathway
Source: Animals (Basel). 2024 Jan 26;14(3):409. doi: 10.3390/ani14030409 (PMC10854683; doi:10.3390/ani14030409)
Supplement: Supplementary file 1 [file animals-14-00409-s001.zip › animals-2799266-supplementary.pdf]

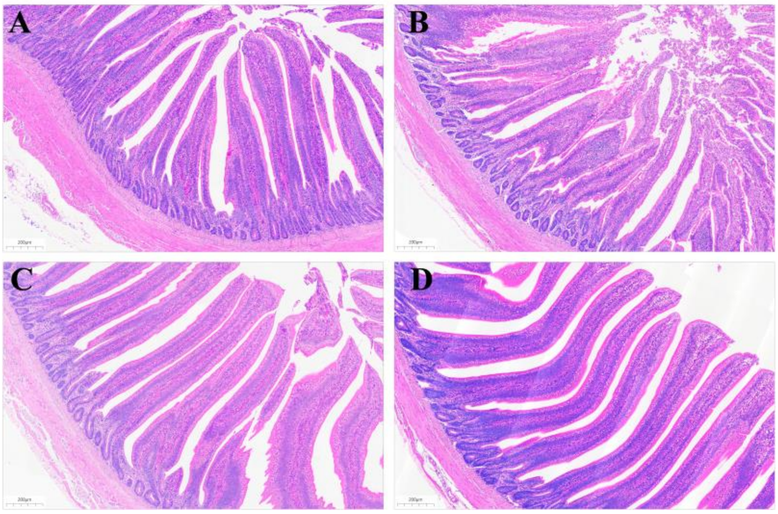

Figure S1. Histological examination broiler ileum (Bar = 200 μm). (**A**) Control group. (**B**) AFB1 group. (**C**) AFB1+CUR group. (**D**) CUR group.
